# Biosynthesis of Camphane Volatile Terpenes in *Amomum villosum* Lour: Involved Genes and Enzymes

**DOI:** 10.3390/plants14121767

**Published:** 2025-06-10

**Authors:** Yuhua Guo, Yamei Li, Pengfei Zhang, Zuliang Luo, Junmei Yin, Xiaojun Ma, Chao Yuan

**Affiliations:** 1 Tropical Crops Genetic Resources Institute, Chinese Academy of Tropical Agricultural Sciences, Haikou 571101, China; guoyuhua01@126.com (Y.G.); lym6137@163.com (Y.L.); pengfeizhang6446@126.com (P.Z.); yinjunmei2004@163.com (J.Y.); 2 Key Laboratory of Crop Gene Resources and Germplasm Enhancement in Southern China, Ministry of Agriculture, Haikou 571101, China; 3 Key Laboratory of Tropical Crops Germplasm Resources Genetic Improvement and Innovation of Hainan Province, Haikou 571101, China; 4 Institute of Medicinal Plant Development, Chinese Academy of Medical Sciences, No. 151 Malianwa North Road, Haidian District, Beijing 100193, China; zuliangluo@163.com

**Keywords:** biosynthesis pathway, camphane volatile terpenes, transcriptome

## Abstract

*Amomum villosum* (*A. villosum*) Lour., a medicinal species of the *Zingiberaceae* family, is used for medical purposes. Bornyl acetate, camphor, and borneol are the main bioactive ingredients in *A. villosum* fruit, and the amount of bornyl acetate is regarded as a measure of the fruit’s quality. In order to explore the major effective genes regulating the biosynthesis of camphane volatile terpenes in *A. villosum,* some DEGs involved in camphane volatile terpene biosynthesis and transcription factors were analyzed and summarized in this study. The result showed that the content of bornyl acetate was altered in the different growth stages. In particular, the significant change occurred from 7 to 30 DAP (days after pollination). The content of bornyl acetate at 30 DAP was 169.3% more than that at 7 DAP. In total, 4782 up-regulated and 5284 down-regulated unigenes were found in G2 vs. G1, as well as 3324 up-regulated and 5036 down-regulated unigenes in G3 vs. G1, and 3332 up-regulated and 4490 down-regulated unigenes in G3 vs. G2. A total of 323 up-regulated and 820 down-regulated unigenes were shared in three growth stage comparisons. We screened the genes that encode the enzymes most likely to inhibit bornyl diphosphate synthase, borneol dehydrogenase, and BAHD acyltransferases. Interestingly, we found that borneol dehydrogenase and bornyl diphosphate synthase displayed bi-substrate features, suggesting that a substrate of catalyzation is promiscuity in the biosynthesis downstream pathway, and the unknown bornyl pyrophosphate hydrolase may not be the specific enzyme for borneol formation. Additionally, the DXR, HDS, and IDS found in the PPI network would assist in the understanding of molecular regulation. The results of this study constructed DGE libraries and identified key genes related to camphane volatile terpenes, which laid a foundation for a deep investigation of the mechanism of volatile terpene biosynthesis, and provided a reference for mining other key genes in *A. villosum* fruits.

## 1. Introduction

*Amomum villosum (A. villosum)* plants, belonging to the Zingiberaceae family, are robust perennial herbs that produce leafy stems that can reach heights of up to three meters from the creeping rhizome [1]. The cultivation of *Amomum villosum* Lour. is a significant form of agroforestry in southern Yunnan and Guangdong, China [2]. The fruit of *A. villosum,* which called Fructus Amomi, has long been used in the Chinese diet, and is officially recognized as “Sha Ren” in the Pharmacopoeia of the People’s Republic of China [3]. The ripe fruit of *A. villosum* has been used in dispelling dampness, treating gastrointestinal diseases, and preventing abortion in China for hundreds of years [4,5,6,7,8]. In recent years, volatile oil and polysaccharides extracted from Fructus Amomi contribute to some important biological functions, such as anti-inflammation, antimicrobial, and antinociceptive activities [9,10,11,12,13]. The volatile oil extracted from the ripe fruits of *A. villosum* contains bioactive compounds such as bornyl acetate (5~47%), camphor (4~17%), borneol (1.5~6%), some primary camphane volatile terpenes, etc. [14]. Among these, bornyl acetate, the key ingredient in essential oils, has long been considered as a standard for the quality of *Fructus Amomi* [15]. The quality of *A. villosum* is usually better than that of *A. longiligulare T. L. Wu* and *A. villosum Lour. var. xanthioides T. L. Wu et Senjen* because of its higher content of volatile oil and bornyl acetate [16]. The demand for *A. villosum* in the domestic market is much greater than that of the latter two, which has long left it in short supply [17]. Therefore, identifying and analyzing the key genes associated with bornyl acetate in *A. villosum,* and elucidating the accumulation rhythm of bornyl acetate in *A. villosum* fruits, will lay a foundation for optimizing the quality of *A. villosum*.

Camphane volatile terpenes are the monoterpenoids and sesquiterpenoids composed of two or three isoprene units, and synthesized by the cyclization of geranyl diphosphate via the isoprenoid pathway to form the primary backbone [18,19]. Information about the upstream biosynthesis of monoterpenoid and sesquiterpenoid skeletons has been reported [20]. This coincides with the triterpenoid biosynthesis pathway, both via two pathways (the mevalonate [MVA] pathway and 2-C-methyl-D-erythritol-4-phosphate [MEP] pathway) to form geranyl diphosphate [21], but several enzymes were characterized in the skeleton biosynthesis of monoterpenoids and sesquiterpenoids, especially the biosynthesis of camphane volatile terpenes, and little about the whole biosynthesis pathway was analyzed. Bornyl diphosphate synthase (BPPS) is an important enzyme in the synthetic pathway of Amomum medicinal terpenoids. Catalyzing geranyl diphosphate (GPP) generates bornyl diphosphate (BPP), which is the direct precursor of the medicinal substance Longnao and the indirect precursor of bornyl acetate in *A. villosum* [22]. To our knowledge, the enzymes characterized in the camphane volatile terpene biosynthesis of A. villosum were 1-deoxy-D-xylulose-5-phosphate reductoisomerase (DXR, EC: 1.1.1.267), 1-deoxy-D-xylulose-5-phosphate synthase (DXS, EC:2.2.1.7), 3-hydroxy-3-methylglutaryl coenzyme A reductase (HMGR, EC:1.1.1.34), and phosphomevalonate kinase (PMK, EC:2.7.4.2) [3,23]. Other enzymes are desired for characterization and analysis. The specific biosynthesis pathway and regulation network of this plant species should be established to meet the development of cultivation as soon as possible.

Currently, research on the biosynthesis of camphene and other volatile terpenes has advanced significantly, particularly due to progress in metabolic engineering and synthetic biology [24]. Engineered E. coli and Saccharomyces cerevisiae have been used to produce camphene [25], but in low yields due to the current challenges of enzyme inefficiency, metabolic burden, and competing pathways (e.g., limonene, pinene). Enzyme engineering is needed to improve the efficiency of camphene synthase [25]. Moreover, there is no molecular information about *A. villosum* in the different development stages, which hinders the progress of research on understanding the biosynthesis process of camphane volatile terpenes.

The development of next-generation sequencing technology (NGS) has made it a potent and affordable tool for identifying novel genes and predicting their functions [26]. Furthermore, RNA-sequencing (RNA-Seq) is known as whole transcriptome shotgun sequencing, overlapping short fragments to cover the whole transcriptome with mechanical mRNA or cDNA fragmenst [27]. It has been widely applied to analyze the regulation of gene expression at a given moment in time in many medicinal plant species, such as *Panax ginseng* [28], *Salvia miltiorrhiza* [29], *Catharanthus roseus* [30], and *Dendrobium officinale* [31]. These findings give better insight for understanding the genes in the secondary metabolite biosynthesis pathway. The quantitative analysis of metabolites is a visualized approach for confirming gene expression. It will be more reliable than using RNA-Seq technology for gene discovery and function prediction in the corresponding crucial development points.

In this study, we constructed six libraries of *A. villosum* at three development stages using RNA-seq technology based on an Illumina HiSeq 4000 platform to provide an entire transcriptomic characterization of its morphology and accumulation of volatile terpenes. The expression of key genes associated with backbone biosynthesis upstream of camphane volatile terpenes that were up- or down-regulated in the RNA-seq experiment, such as *DXR*, *DXS*, and *PMK*, were verified by real-time RT-PCR. Otherwise, some genes that are most likely to be involved in biosynthesis downstream of camphane volatile terpenes were screened; transcriptome factors possibly related to the biosynthesis of the secondary metabolite were selected, classified, and predicted. Protein–protein interaction was performed by visualization in Cytoscape V3.6.1 in three development stages. Therefore, the transcriptome databases contribute to understanding the biosynthesis of camphane volatile terpenes, and may provide a good foundation for further research on the molecular regulation mechanisms associated with biosynthesis and the emission of volatile terpenes.

## 2. Results

### 2.1. Morphological Characteristics and Quantitative Analysis of Bornyl Acetate of A. villosum Fruits

**Figure 1:**
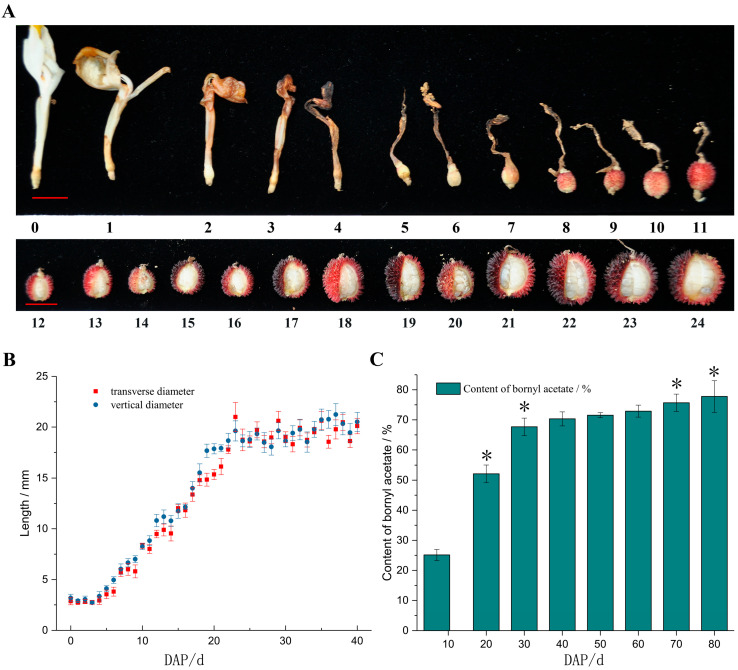
Changes in *Amomum villosum* (*A. villosum*) Lour. fruit at different growing stages. (**A**) Development of *A. villosum* 24 days after pollination. The figures below the picture correspond to the sampling time. Red scale bar, 1 cm. (**B**) Changes in fruit diameter of *A. villosum*. (**C**) The relative content percentage of bornyl acetate in *A. villosum* at different growth stages. Values are shown as the mean ± SD (*n* = 3). Note: * indicates significant differences in the index values of various treatment durations (*p* < 0.05).

The morphological changes of *A. villosum* fruits were observed from 0 to 24 days after pollination (DAP), and obvious growth in the fruits was observed from 5 to 20 DAP (Figure 1A). When the transverse diameter and vertical diameter of the fruits was measured, both the transverse and vertical diameter growth rates were nearly the same. They were prone to stabilization after 25 DAP (Figure 1B).

The bornyl acetate content in *A. villosum* fruits at 7, 20, 30, 40, 50, 60, 70, and 80 DAP was evaluated by GC-MS analysis. The results of bornyl acetate accumulation are shown in Figure 1C. The content of bornyl acetate was altered in the different growth stages. In particular, the significant change occurred from 7 to 30 DAP (*p* < 0.05). The content of bornyl acetate at 30 DAP was 169.3% more than that at 7 DAP. With a longer growth time (30–80 DAP), bornyl acetate accumulates at a smaller rate (Figure 1C), implying that a good collection time for *A. villosum* fruits should set at after the 30th day, and that good approaches to molecular regulation should also be carried out on time.

### 2.2. Differentially Expressed Gene (DEG) Identification

**Figure 2:**
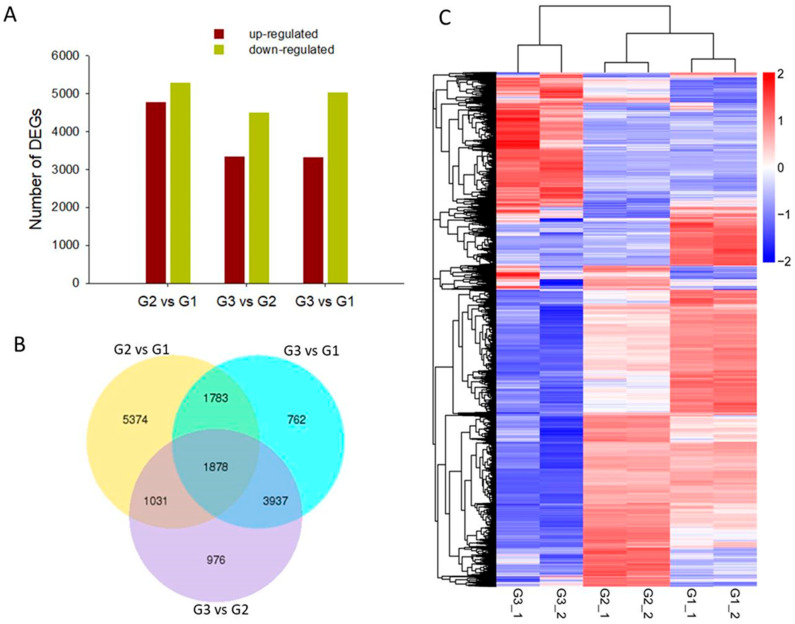
Overview of transcriptome at different growing stages of fruit (**A**) Number of differentially expressed genes (DEGs) (log2 Foldchange ≥ 1 and adjusted *p* < 0.05, *p*-value was adjusted using *q*-value). (**B**) Distribution of DEGs across the three developmental stages. (**C**) Heatmap showing the cluster analysis of all DEG expression profiling.

DEGs with a false discovery rate (FDR, *q*-value) < 0.005 and |log2 (fold change)| > 1 were used to explore gene expression during different growth stages of *A. villosum* fruit. In the results of the DEG analysis, we identified 10,066 DEGs in a group of G2 vs. G1, with 4782 up-regulated and 5284 down-regulated unigenes. In G3 vs. G2, 7822 genes were expressed at significantly different levels (3332 up-regulated and 4490 down-regulated). Furthermore, 8360 DEGs were observed in G3 vs. G1 (3324 up-regulated and 5036 down-regulated) (Figure 2A). Furthermore, 1878 DEGs were shared in three comparable groups according to an analysis of Venn diagrams (Figure 2B), and a cluster analysis of all DEG expression was shown in a heatmap (Figure 2C).

### 2.3. Functional Classification of the DEGs

**Figure 3:**
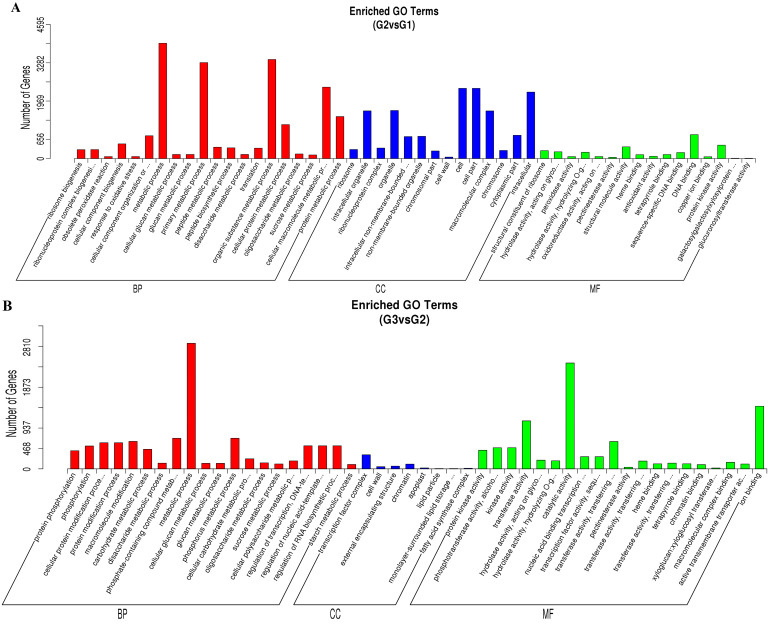

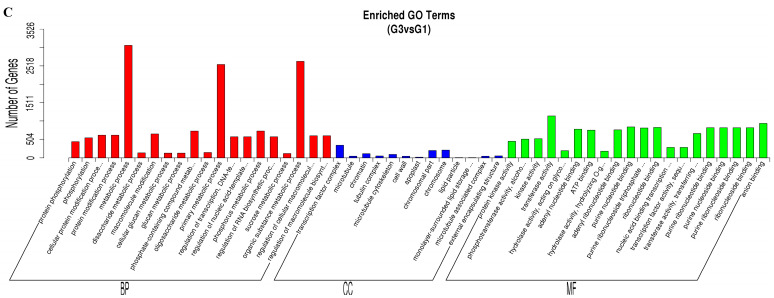
GO functional classification of DEGs. (**A**) G2 vs. G1. (**B**) G3 vs. G2. (**C**) G3 vs. G1.

After a GO enrichment analysis of the DEGs, there were 76, 134, and 126 enriched GO terms, consisting of various biological processes in the three comparisons of G2 vs. G1, G3 vs. G2, and G3 vs. G1, respectively (Figure 3). There were 46, 14 and 16 terms in BP, CC, and MF functional ontologies in G2 vs. G1, respectively. Many of the enriched terms were found in the metabolism and cellular component, including ‘metabolic process’ (GO:0008152), ‘organic substance metabolic process’ (GO:0071704), ‘primary metabolic process’ (GO:0044238), ‘cell’ (GO:0005623), ‘cell part’ (GO:0044464), and ‘intracellular’ (GO:0005622), which correspond to the results of the morphology changes. In contrast, a very high number of DEGs enriched GO terms for G3 vs. G2 and G3 vs. G1, which consist of ‘metabolic process’ (GO:0008152), ‘organic substance metabolic process’ (GO:0071704), ‘primary metabolic process’ (GO:0044238), ‘catalytic activity’ (GO:0003824), and ‘ion binding’ (GO:0043167) (Table S1). This implied that secondary metabolites, including bornyl acetate, were mainly accumulated in the later stage, which agreed with the findings of the bornyl acetate content analysis of *A. villosum* fruits at various growth stages.

**Figure 4:**
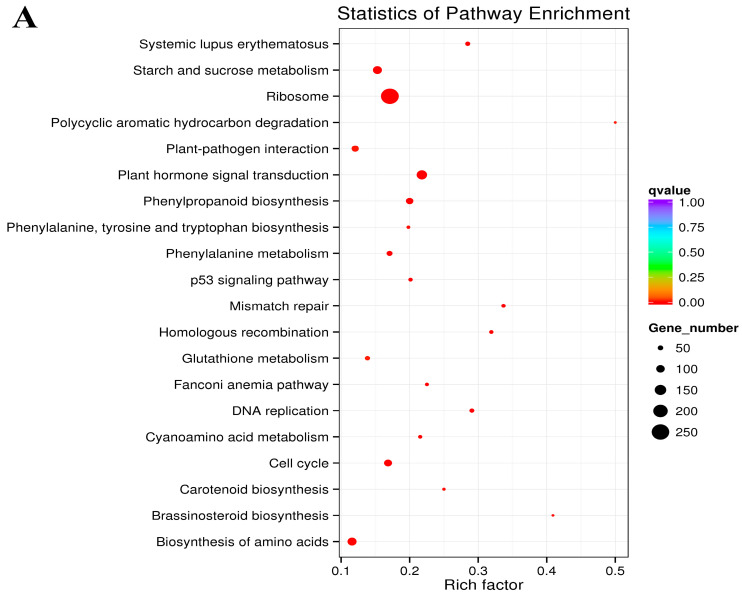

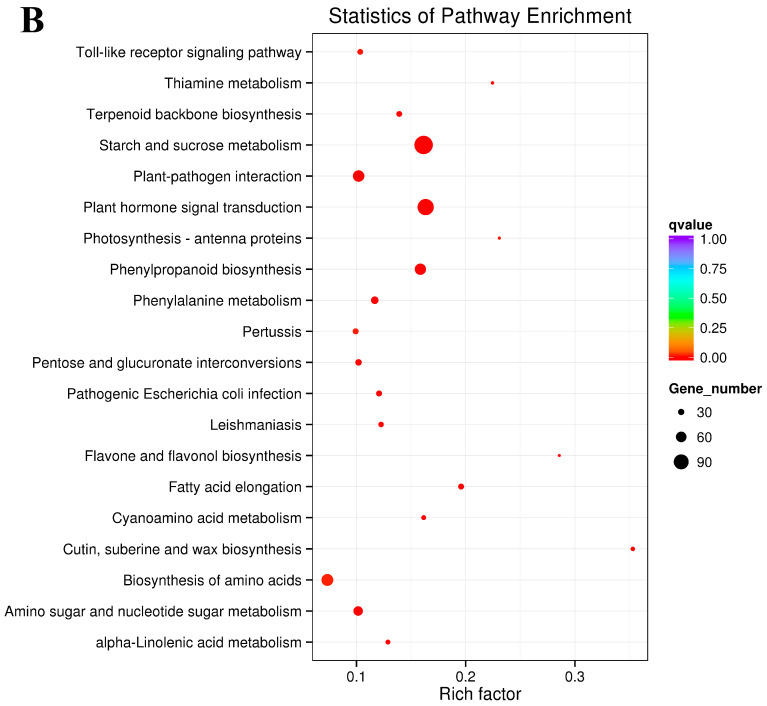

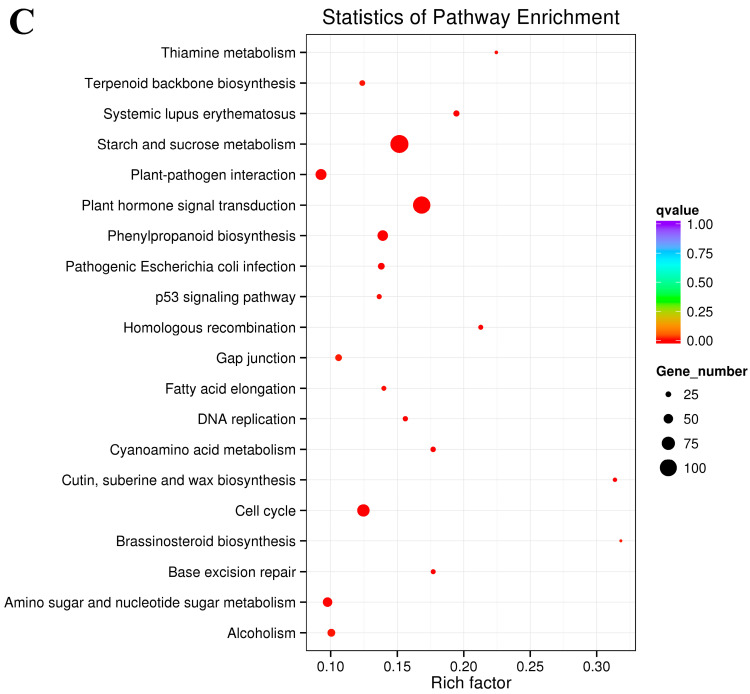
KEGG pathway enrichment analysis of DEGs. (**A**) G2 vs. G1. (**B**) G3 vs. G2. (**C**) G3 vs. G1.

The biological functions of various genes were coordinated in vivo, and pathway substantial enrichment allowed the identification of the key biochemical metabolic and signal transduction pathways involved in differentially expressed genes. Overall, KEGG pathway enrichment analysis revealed 40, 35 and 35 pathways in G2 vs. G1, G3 vs. G2, and G3 vs. G1, respectively (Table S2). The pathways in G2 vs. G1 were mainly involved in primary metabolite biosynthesis and metabolism, such as ‘biosynthesis of amino acids’ (ko01230), ‘phenylalanine, tyrosine, and tryptophan biosynthesis’ (ko00400), ‘starch and sucrose metabolism’ (ko00500), and the signaling transduction pathway ‘plant hormone signal transduction’ (ko04075). In addition, we found several pathways associated with secondary metabolites both in G3 vs. G2 and G3 vs. G1. Besides the primary metabolite pathways, ‘terpenoid backbone biosynthesis’ (ko00900) and ‘flavone and flavonol biosynthesis’ (ko00944) were found in both. After comparation of G3 vs. G1 and G3 vs. G2, ‘steroid biosynthesis’ (ko00100) and ‘brassinosteroid biosynthesis’ (ko00905) were found in G3 vs. G1, but ‘tropane, piperidine and pyridine alkaloid biosynthesis’ (ko00960) and ‘diterpenoid biosynthesis’ (ko00904) were found in G3 vs. G2 (Figure 4). All these results suggest that the genes involved in camphane volatile terpene biosynthesis might show significant expression in later stages.

### 2.4. Network Analysis of Protein Interaction Based on the DEGs

**Figure 5:**
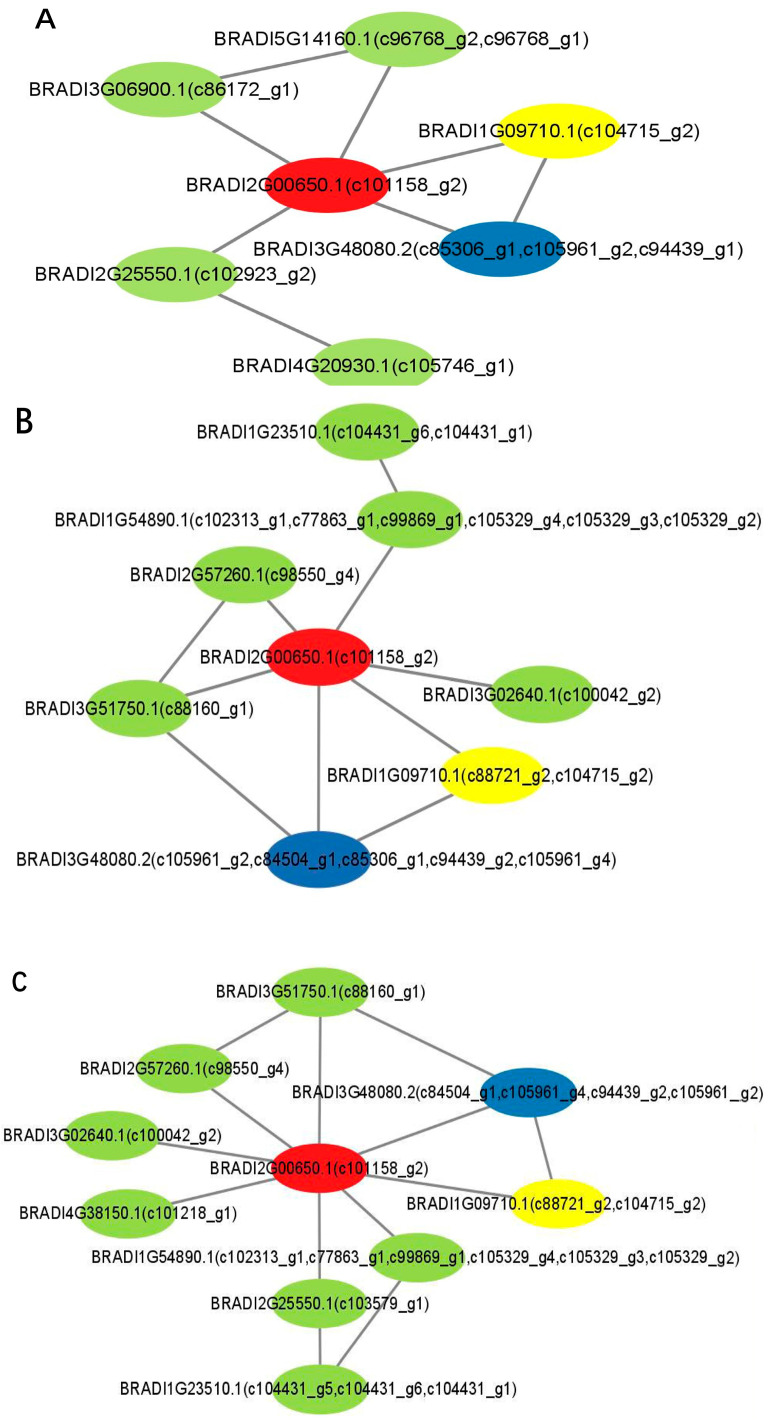
Network analysis of protein interaction based on the DEGs. (**A**) G2 vs. G1. (**B**) G3 vs. G1. (**C**) G3 vs. G2. Red nodes represent DXR, yellow IDS, and blue HDS.

Protein–protein interaction (PPI) was significant in the biological process. In this PPI network, node represents protein, which results from the DEG sequence blastx to the genome of a relevant species. The PPI was defined as the edge, and a number of protein interactions with other proteins were defined as the degree, which positively correlate with the intermediate centrality of the node. According to the results of blastx to the STRING database, the PPI network of DEGs in G2 vs. G1 consisted of 365 nodes and 1218 edges, and the highest degree was 80. The PPI network of DEGs in G3 vs. G1 comprised 173 nodes and 476 edges, whereas 72 nodes and 60 edges were found in the PPI network of DEGs in G3 vs. G2. Fortunately, 1-deoxy-D-xylulose-5-phosphate reductoisomerase (DXR), 4-hydroxy-3-methylbut-2-en-1-yl diphosphate synthase (HDS), and 4-hydroxy-3-methylbut-2-en-1-yl diphosphate reductase (IDS) were found in the three PPI networks. These were the key enzymes in the skeleton formation of terpenoids. Furthermore, various types of ribosomal protein and chaperone were involved in the PPI network, which implied that they might take active roles in the regulation of biological processes (Figure 5).

### 2.5. Unigenes Involved in Backbone Biosynthesis of Camphane Volatile Terpenes

**Figure 6:**
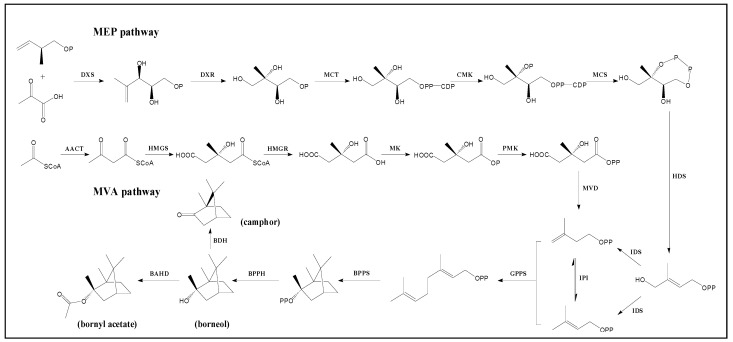
Putative amphane volatile terpene biosynthesis pathway in *Amomum villosum* Lour. DXS: 1-deoxy-D-xylulose-5-phosphate synthase, EC: 2.2.1.7; DXR: 1-deoxy-D-xylulose-5-phosphate reductoisomerase, EC:1.1.1.267; MCT: 2-C-methyl-D-erythritol 4-phosphate cytidylyltransferase, EC:2.7.7.60; CMK: 4-(cytidine 5′-diphospho)-2-C-methyl-D-erythritol kinase, EC:2.7.1.148; MCS: 2-C-methyl-D-erythritol 2,4-cyclodiphosphate synthase, EC:4.6.1.12; HDS: 4-hydroxy-3-methylbut-2-en-1-yl diphosphate synthase, EC:1.17.7.1; IDS: 4-hydroxy-3-methylbut-2-en-1-yl diphosphate reductase, EC:1.17.1.2; AACT: acetyl-CoA acetyltransferase, EC:2.3.1.9; HMGS: 3-hydroxy-3-methylglutaryl coenzyme A synthase, EC:2.3.3.10; HMGR: 3-hydroxy-3-methylglutaryl coenzyme A reductase, EC:1.1.1.34; MK: mevalonate kinase, EC:2.7.1.36; PMK: phosphomevalonate kinase, EC:2.7.4.2; MVD: diphosphomevalonate decarboxylase, EC:4.1.1.33; IPI: isopentenyl-diphosphate Delta-isomerase, EC:5.3.3.2; GPPS: geranyl-diphosphate synthase, EC:2.5.1.1; BPPS: bornyl diphosphate synthase, EC: 5.5.1.8; BPPH: bornyl pyrophosphate hydrolase, EC: 3.1.7.3; BDH: borneol dehydrogenase, EC: 1.1.1.198; BAHD: BAHD acyltransferase, EC: 2.3.1.

The isoprenoid route is used to create camphane volatile terpenes (mainly involving monoterpenes and sesquiterpenes) by cyclizing geranyl diphosphate, primarily to generate the backbone. After that, the backbone goes through a number of alterations (oxidation, reduction, and substitution), which are aided by a number of enzymes, e.g., geranyl diphosphate, dehydrogenase and acyltransferases, to create the volatile terpene [32] (Figure 6). Evidence has shown that both the cytosolic MVA pathway and the plastidial MEP pathway can produce isopentenyl diphosphate (IPP) in the early stage of active isoprene unit formation, which is then converted to its allylic isomer [33]. The upstream genes in the backbone biosynthesis of camphane volatile terpenes were mapped in the KOG classification. Bornyl diphosphate synthase (BPPS), bornyl pyrophosphate hydrolase (BPPH), borneol dehydrogenase (BDH), and BAHD acyltransferases in the downstream need further research for screening. A wide family of enzymes known as BAHD acyltransferases is responsible for the acyl-CoA dependent acylation of secondary metabolites, which commonly produces esters and amides [34]. The family name of BAHD acyltransferases is composed of the first four characterized members (BEAT or benzylalcohol O-acetyltransferase from Clarkia breweri; AHCTs or anthocyanin O-hydroxycinnamoyltransferases from Petunia, Senecio, Gentiana, Perilla, and Lavandula; HCBT or anthranilate N-hydroxycinnamoyl/benzoyltransferase from Dianthus caryophyllus; and DAT or deacetylvindoline 4-O-acetyltransferase from Catharanthus roseus) [35,36]. It has been reported that monoterpene acetates were produced by acetyltransferase in *Lavandula x intermedia* [37]. Fortunately, three candidate *BDHs* (unigene c96075_g1, unigene c105839_g1, and unigene c85128_g2) were found to be close to genes encoding a short-chain dehydrogenase in *Artemisia annua* and *L. x intermedia*. The sequence of unigene c105839_g1 was blasted to the National Center for Biotechnology Information (NCBI) and corresponded with the *Zingiber zerumbet zsd1* mRNA for short-chain dehydrogenase/reductase1 (accession: AB480831.1) [38]. Nine candidate *BPPSs* (unigene c103210_g1, unigene c95418_g1, unigene c102189_g1, unigene c46547_g1, unigene c105905_g3, unigene c82785_g1, unigene c98804_g1, unigene c91238_g1, and unigene c106371_g2) were matched with genes encoding the bornyl diphosphate synthase in *Salvia officinalis* and *Lavandula angustifolia* subsp [22]. Moreover, seven unigenes (unigene c160888_g1, unigene c76165_g1, unigene c199426_g1, unigene c150006_g1, unigene c92359_g2, unigene c23572_g1, and unigene c33340_g2) were annotated with BAHD acyltransferases, but only unigene c92359_g2 belongs to the DEGs. However, unigenes involved in the BPPH were not found in the sequence annotation against seven databases, and little research was available for reference. The process transforming bornyl diphosphate to bornyl acetate might be intricate.

**Figure 7:**
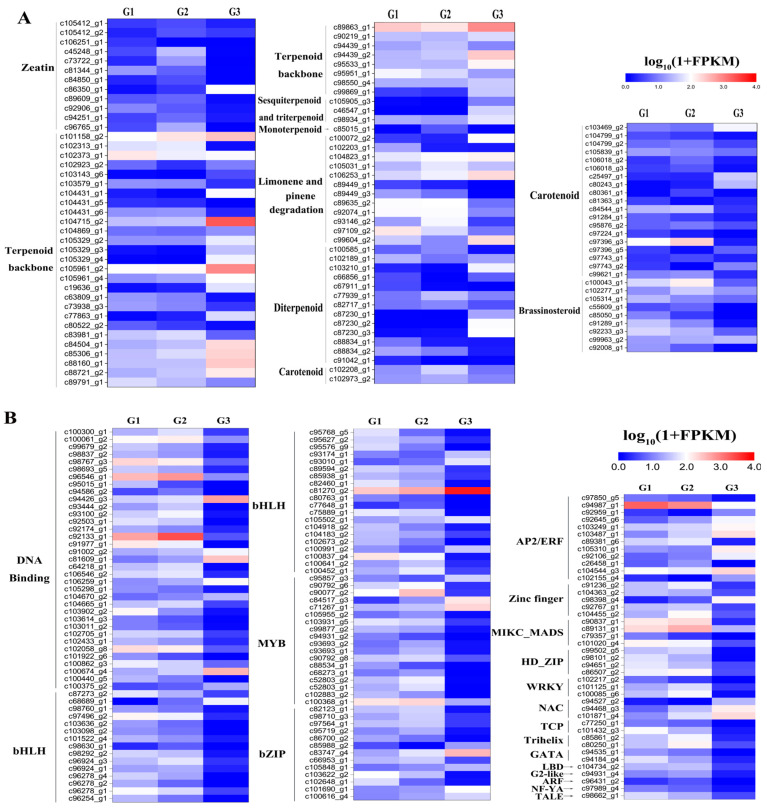
Expression profile of DEGs in terpenoid and polyketide biosynthesis pathways and putative transcription factors. (**A**) Expressions of DEGs associated with biosynthesis pathways of terpenoids and polyketides. (**B**) Expressions of DEGs involved in transcription factors.

In the KOG classification of DEGs, 106 DEGs were assigned to eight biosynthesis pathways for terpenoids and polyketides. The largest subcategory was ‘Terpenoid backbone biosynthesis’ (35), followed by ‘Carotenoid biosynthesis’ (21), ‘Limonene and pinene degradation’ (12), and ‘Zeatin biosynthesis’ (12) (Figure 7A). Excitingly, 32 DEGs in the ‘Terpenoid backbone biosynthesis’ mapped into the backbone biosynthesis of camphane volatile terpenes (Table S3). The expression of DEGs in the backbone biosynthesis pathway of camphane volatile terpenes might account for the regulation of transcription factors (TFs). Furthermore, we analyzed the DEGs annotated in the transcription factor. In total, 143 DEGs of TFs were divided into 18 subfamilies, including MYB, bHLH, AP2/ERF, WRKY, bZIPs, and so on (Figure 7B). According to the findings, several TFs engaged in the biosynthesis of terpenoids that were downregulated in G3 may be in conflict with the expression of genes involved in the production pathway of terpenoids.

### 2.6. Verification of RNA-Seq Data by qRT-PCR

**Figure 8:**
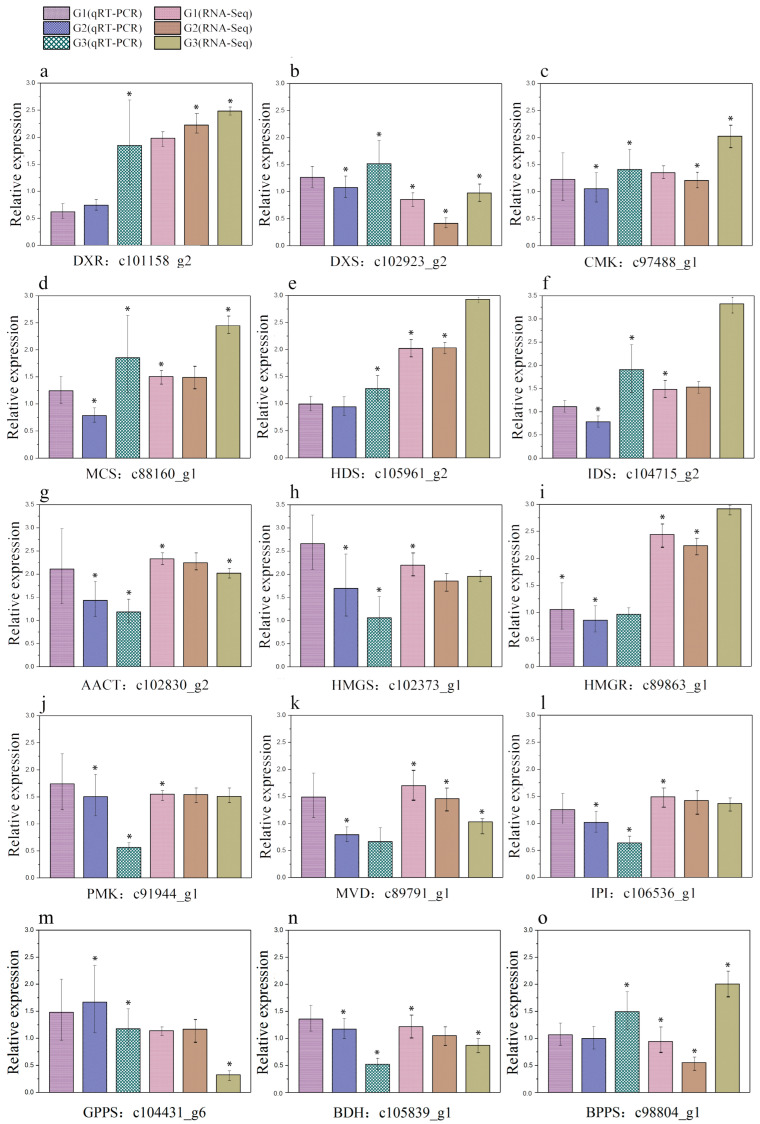
Validation of the RNA-Seq results with qRT-PCR experiments. Fifteen unigenes associated with the biosynthesis of camphane volatile terpenes were selected and subjected to qRT-PCR analysis. The expression levels between RNA-Seq and qRT-PCR experiments for these unigenes are shown in panels (**a**–**o**), respectively. The error bar for the qRT-PCR results corresponds to the variations among the three biological replicates and three technical replicates. The value of r is the Pearson’s correlation coefficients between the expression profiles obtained from the RNA-Seq and qRT-PCR experiments. Note: * indicates significant differences in the index values of various treatment durations (*p* < 0.05).

The qRT-PCR experiment was performed in three different growing stages due to the high throughput, sensitivity, and accuracy of the RNA-Seq data. In this study, the DEGs chosen for this comparison are those unigenes putatively involved in the biosynthesis of camphane volatile terpenoids. The expression levels from the RNA-Seq and qRT-PCR studies were normalized against the average expression levels from the three stages for each transcript after being log transformed. Otherwise, we calculated the Pearson’s correlation coefficients (r) between the expression profiles obtained from the RNA-Seq and qRT-PCR experiments. In particular, 11 out of the 15 DEGs (about 73.33%) had *r* values ≥ 0.90 (Figure 8, Table S4). In other words, the results from the RNA-Seq experiments correlated well with those of the RT-qPCR experiments, indicating that our experimental results were reliable.

## 3. Discussion

Volatile terpenes (monoterpene and sesquiterpene), not separate monomers, are the medicinal ingredient in Chinese medicine. Fructus Amomi, which contains volatile terpenes such as bornyl acetate, borneol, and camphor, belongs to the homogeneous medicine and food that is prevalent in Southeast Asia. Because of the length of the harvest (July to September) and lack of light and temperature, some fruits cannot mature completely. Provided that the fruits at different stages of ripening are mixed with unripe fruits, they cannot avoid the influence of the efficacy of Fructus Amomi. In this study, morphological changes and fruit size were measured, and quantitative analyses of bornyl acetate at different growth stages were established by GC-MS. The results demonstrate that the increase in weight and volume of *A. villosum* fruits occurs on the 5th–24th day (Figure 1A,B), and tends to stabilize after 24 days. The content of bornyl acetate was altered in the different growth stages. The significant change occurred from 7 to 30 DAP (*p* < 0.05), in particular. The content of bornyl acetate at 30 DAP increased sharply, then remained stable up to the 80th day (Figure 1C). From the results, we could clearly discriminate the timing difference of bornyl acetate in fruits at different development stages, and we could provide scientific evidence for timing the fruit harvest. Furthermore, it was significant that we could further research the molecular regulatory mechanism to improve volatile terpene yield in the crucial stages of fruit development.

De novo transcriptome assembly of RNA-Seq involves the full transcriptome, and screening of genes with different expression levels could be conducted to explore obscure biological process [39,40]. Transcriptome profile analysis is an effective approach to obtain functional genes and regulation pathways [41], such as some genes and pathways involved in the response to water loss in *Taxillusi chinensis* (DC.) Dansers were identified [42]. To fully regulate the gene expression of *A. villosum* fruits in different growth stages, several transcriptomes were carried out in materials with different treatments [43,44]. However, significant progress has not yet been made in molecular research on this plant species. Only a few key genes involved in the biosynthesis of camphane volatile terpenes have been characterized and functionally analyzed, and knowledge of the whole process of volatile terpene biosynthesis is incomplete. Therefore, it is desirous to acquire further information on various modifications in the biosynthesis pathway downstream. In the study, we obtained three libraries with two biological repeats using the Illumina 4000 platform, producing 214,320 unigenes, which is larger than the previous transcriptome databases. As shown in this study, a large number (135 and 62 respectively) of screening genes were produced based on sequence alignment with *BDH* and *BPPS* in *S. officinalis* and *L. angustifolia* subsp. All of them might play roles in the catalytic reactions of camphane volatile terpene backbone formation at different times, but usually several of them are crucial. The labor required to characterize the enzymes actually engaged in this pathway increases due to the increased screening findings. Three *BDHs* and nine *BPPSs* were selected as the candidates most likely associated with volatile terpene biosynthesis through analysis of the sequence alignment and different expressions of unigenes at three different development stages. RNA-Seq combined with different expressions is an effective tool for the functional gene excavation of the response under adversity stress [45,46], the secondary metabolism pathway [47,48], the metamorphosis process [49], etc. The transcriptome on the different development stages in the fruit of *A. villosum* upgraded the transcriptome resources and is regarded as a significant cornerstone in the further investigation of the secondary metabolite biosynthesis pathway. These candidates’ functions may be verified using in vitro enzymatic assays and heterologous expressions in yeast or *Escherichia coli*.

In this study, although the transcriptome of *A. villosum* fruit offered a highly effective technique to find the genes linked to recognized enzymes involved in the production of secondary metabolites, as well as candidate genes that might be linked to as yet unidentified steps in the pathway (Table S3), no DEGs were found to be involved in the 4-(cytidine 5′-diphospho)-2-C-methyl-D-erythritol kinase (CMK), acetyl-CoA acetyltransferase (AACT), PMK and isopentenyl-diphosphate Delta-isomerase (IPI), and unigenes associated with BPPH, and also were not found in the sequence annotation. It is difficult to screen the enzymes actually involved in the downstream pathway of volatile terpene biosynthesis. BPPS belongs to the family of terpenoid cyclases (TPSs), and is responsible for the cyclization of geranyl diphosphate to generate (+)-bornyl diphosphate, which is vital for the cyclizing process in monoterpenoid biosynthesis [50]. It was found in *Salvia officinalis* that (+)-bornyl diphosphate synthase might represent both (+)-bornyl diphosphate synthase and (+)-pinene synthase, which were previously assumed to be distinct enzymes [51]. BDH, an enzyme that catalyzes borneol to camphor, was named as a short-chain dehydrogenase in *Zingiber zerumbet*, which belongs to the short-chain dehydrogenase/reductase superfamily (SDR), displaying a bisubstrate feature corresponding with its flexible substrate-binding pocket [37,38]. Moreover, it was reported that (+)-(3S)-neomenthol reductase could catalyze different terpene products (such as (+)-(3S)-neomenthol, (–)-(3R)-menthol, (+)-(3S)-isomenthol, and (+)-(3R)-neoisomenthol), and the diversity of substrates and biosynthesis results in more than one product catalyzed by terpene synthase [52]. TPSs are responsible for the initial cyclization cascade in the multistep synthesis of more than 60,000 known natural compounds, and due to the extremely small pool of substrates based on linear isoprenoids, these are often promiscuous and should face the challenge of a tremendous catalytic reaction [53]. Some TPSs with diverse domains might sometimes take the place of the catalytic function of known enzymes. Moreover, using de novo assembly without a reference genome, short transcripts or low expression genes may not be correctly spliced. The expression level is low. If the expression level of these enzyme genes is lower than the technical detection value (such as FPKMIRPKMITPM < 0.1), they may be filtered out or marked as “not expressed”. The counts/TPM values of these genes in the original expression matrix will be checked, or their expression will be verified by qPCR. These might illustrate why no DEGs were found in some genes and BPPH was not obtained from the database. Incomplete annotations can be supplemented by homologous gene alignment (BLAST, https://blast.ncbi.nlm.nih.gov/Blast.cgi, 7 June 2024) or de novo prediction.

It is acknowledged that the expression of genes in the plant secondary metabolism pathway is regulated by various transcription factors [54,55]. Moreover, several types of transcription factors belonging to the families bHLH, MYB, AP2/ERF, and WRKY have been identified in the regulation of secondary metabolism pathway in plants [56,57,58,59]. In this analysis, a total of 143 unigenes were found in the DEGs. The top three were the families of bHLH (24.5%), MYB (12.6%), and bZIP (9.1%), which is almost in agreement with the results of one report [44]. The bHLH and MYB have been reported as master regulators in the terpenoid biosynthesis pathway [60]. It is possible that some TFs that we classified were involved in the regulation of volatile terpene biosynthesis in *A. villosum*. Further research should be carried out to verify these inferences.

In the analysis of the PPI network, we found many types of ribosomal proteins in the G2 vs. G1 and G3 vs. G1 groups, implying that they might participate in physiological and biochemical processes such as cell expansion and differentiation in the G1 period. Many interactive proteins associated with the secondary metabolism pathway were shown in the G3 vs. G2 PPI network, indicating that this is a significant period for biosynthesis and the accumulation of secondary metabolites. These results corresponded with the morphological characteristics of *A. villosum* in development. Furthermore, the PPI information about *DXR* (unigene c101158_g2), *HMGS* (unigene c102373_g1), *MK* (unigene c73938_g3), and *MCS* (unigene c88160_g1) were available for further research on the regulation to improve the medicinal quality of *A. villosum*.

## 4. Materials and Methods

### 4.1. Collection and Preparation of Samples

The *A. villosum* fruits are picked 70–80 days after pollination (DAP) for medicinal use. Accordingly, at least six biological replicates of plant samples were collected from 0 DAP to 80 DAP from the Botanical Garden of Xishuangbanna South Medicine, Jinghong, China. All the plants and their fruits used in this study were bred and identified by Dr. Xiaojun Ma. Afterward, all samples were divided into eight groups, including those picked 7, 20, 30, 40, 50, 60, 70, and 80 DAP, and all groups with each replicate consisting of pooled fruit tissues from three individual plants were used for chemical quantitative analysis research. Fruits picked 7, 20, and 40 DAP were chosen for molecular research with two biological repeats. The plant tissues were divided into pieces, immediately frozen in liquid nitrogen, and kept in a refrigerator at −80 °C for further research.

### 4.2. Growth Responses and Content Determination of Bornyl Acetate in A. villosum

Using a vernier caliper to measure the transverse diameter and vertical diameter with three biological replicates in fresh fruit picked 0 to 40 DAP, the growth changes of fruits picked 24 DAP were noted using photographs. All samples for content determination were dried in room temperature, completely mixed, and ground into powder. An amount of 0.50 g of sample powder was used for steam distillation, and according to the PPRC (2015) [15], the sample preparation for the gas chromatography analysis was carried out. Chromatography analyses were performed using Shimadzu GC/MS-QP 5000 (Shimadzu Corporation, Shimadzu, Japan) with a Supelco fused silica capillary column DB-1. The carrier gas used a 1.3 mL/min carrier flow rate. All the samples were analyzed in full scan ion monitoring mode. The National Institutes for Food and Drug Control in Beijing, China supplied the bornyl acetate standard material.

### 4.3. Construction of Libraries, Sequence Reads Mapping, Assembly and Annotation

Fruit samples of *A. villosum* at three developmental stages (7, 20, and 40 DAP, designated as G1, G2, and G3) were collected. Total RNA was extracted using Trizol reagent (Cat. No. 15596-026, 15596-018; Thermo Fisher Scientific Inc., Carlsbad, CA, USA) following the manufacturer’s protocol. For each sample, 1.5 μg of RNA with acceptable integrity (RIN) was used as input material. Two biological replicates per stage (total of six samples) were used for library construction.

The clear, high-quality readings served as the foundation for all downstream studies. The left files (read1 files) and right files (read2 files) from all samples were combined into two large files, i.e., left.fq and right.fq. The constructed unigenes were analyzed using BLAST (E value < 10^−5^) analysis against NCBI non-redundant protein (NR), Swiss-Prot, Gene Ontology (GO), Clusters of Orthologous Groups (COG) and the Kyoto Encyclopedia of Genes and Genomes (KEGG).

### 4.4. Gene Expression

The different expression genes (DEGs) were estimated as fragments per kilo of the transcript base per million mapped reads (FPKM) [61,62]. Using the DESeq R package (1.10.1), differential expression analysis was carried out [63]. The GOseq R packages used Wallenius non-central hyper-geometric distribution to perform GO enrichment analysis of DEGs [64,65]. The online database called KEGG [66] is used to understand and to study the high-level uses and functions of biological systems (http://www.genome.jp/kegg/ (6 July 2024)). The KO-Based Annotation System 3.0 (KOBAS3.0) software was used to examine the statistical enrichment of the DEGs in KEGG pathways [67].

The STRING database (http://string-db.org/), which contains 9,600,000 different types of protein in 2000 different organisms, has the protein interaction (PPI) that was used to predict the PPI of these DEGs with an E-value  <  0.05. Cytoscape was then used to display the PPI of these DEGs [68].

### 4.5. RT-qPCR Validation

Total RNA of the samples was extracted with the method described above and stored at −80 °C. TransScript One-Step gDNA Removal and cDNA Synthesis SuperMix were used to reverse transcribe the RNA based on the manufacturer’s protocol (Transgen Biotech, Beijing, China) [69]. All the primers were listed in Table S5 and designed using the Primer Premier 5.0 software.

The reaction procedure was as follows: pre-denaturation at 95 °C for 30 s, followed by 40 cycles of denaturation at 95 °C for 5 s, and annealing/extension at 60 °C for 30 s. All reactions were performed in triplicate. The relative transcript level of each gene was quantified using the Ct value obtained from the reaction [70]. The housekeeping gene ef-1α was used as an internal control [71,72].

## 5. Conclusions

In conclusion, gene analysis and morphological observation reveal that the traits of *A. villosum* fruit varied, and accumulated bornyl acetate depending on the stage of growth. The content of bornyl acetate at 30 DAP had accumulated sharply, with 169.3% more than that at 7 DAP. Using the Illumina 4000 RNA-Seq system, the molecular mechanism was created based on the transcriptome of the fruits of *A. villosum* at various developmental phases. Our study recorded a total of 322,542,986 clean reads, which were assembled into 214,320 unigenes, and which supplement the previous database. From this database, genes involved in the biosynthesis of the backbone of volatile terpenes were identified, and some of their DEGs were validated with qRT-PCR. Moreover, the enzyme-encoding genes, e.g., catalyze, in the putative biosynthesis pathway downstream were screened, and the DEG annotated transcription factors were analyzed with different families. Furthermore, by analyzing the results of visualization in Cytoscape, PPI networks were shown in detail, and DXR, HDS, and IDS were found in the network. Our findings revealed differentially expressed genes across various fruit developmental stages of *A. villosum*, as well as genes associated with the synthesis of camphene volatile terpenes. Nevertheless, further research is needed, including in-depth investigation and identification of key biosynthetic genes of other terpenes.

## Data Availability

Data will be made available on request.

## Supplementary Materials

The following supporting information can be downloaded at: https://www.mdpi.com/article/10.3390/plants14121767/s1.

## Abbreviations

The following abbreviations are used in this manuscript:

DEGs	different expression genes
BAHD	family of enzymes found primarily in plants
DXR	1-deoxy-D-xylulose-5-phosphate reductoisomerase
HDS	4-hydroxy-3-methylbut-2-en-1-yl diphosphate synthase
IDS	diphosphate reductase
PPI	protein–protein interaction
