# Supplementary material for: Biosynthesis of Camphane Volatile Terpenes in Amomum villosum Lour: Involved Genes and Enzymes
Source: Plants (Basel). 2025 Jun 10;14(12):1767. doi: 10.3390/plants14121767 (PMC12197307; doi:10.3390/plants14121767)
Supplement: Supplementary file 1 [file plants-14-01767-s001.zip › Table A3.pdf]

Table A3| The numbers of unigenes involved in camphane volatile terpenes biosynthesis

Gene	Enzyme No.	No. of annotation	No. of DEGs
1-deoxy-D-xylulose-5-phosphate synthase (DXS)	EC:2.2.1.7	23	9
1-deoxy-D-xylulose-5-phosphate reductoisomerase (DXR)	EC:1.1.1.267	4	1
2-C-methyl-D-erythritol 4-phosphate cytidylyltransferase (MCT)	EC:2.7.7.60	1	1
4-(cytidine 5'-diphospho)-2-C-methyl-D-erythritol kinase (CMK)	EC:2.7.1.148	4	0
2-C-methyl-D-erythritol 2,4-cyclodiphosphate synthase (MCS)	EC:4.6.1.12	2	1
4-hydroxy-3-methylbut-2-en-1-yl diphosphate synthase (HDS)	EC:1.17.7.1	18	6
4-hydroxy-3-methylbut-2-en-1-yl diphosphate reductase (IDS)	EC:1.17.1.2	7	2
acetyl-CoA acetyltransferase (AACT)	EC:2.3.1.9	10	0
3-hydroxy-3-methylglutaryl coenzyme A synthase (HMGS)	EC:2.3.3.10	4	1
3-hydroxy-3-methylglutaryl coenzyme A reductase (HMGR)	EC:1.1.1.34	17	3
mevalonate kinase (MK)	EC:2.7.1.36	8	2
phosphomevalonate kinase (PMK)	EC:2.7.4.2	3	0
diphosphomevalonate decarboxylase (MVD)	EC:4.1.1.33	5	1
isopentenyl-diphosphate Delta-isomerase (IPI)	EC:5.3.3.2	8	0
geranyl-diphosphate synthase (GPPS)	EC:2.5.1.1	25	5
bornyl diphosphate synthase (BPPS)	EC:5.5.1.8	9	9
bornyl pyrophosphate hydrolase (BPPH)	EC:3.1.7.3	0	0
borneol dehydrogenase (BDH)	EC:1.1.1.198	3	3
borneol: acetyl coenzyme A acetyltransferase (BAHD)	EC:2.3.1.-	7	1
