# Supplementary material for: Biosynthesis of Camphane Volatile Terpenes in Amomum villosum Lour: Involved Genes and Enzymes
Source: Plants (Basel). 2025 Jun 10;14(12):1767. doi: 10.3390/plants14121767 (PMC12197307; doi:10.3390/plants14121767)
Supplement: Supplementary file 1 [file plants-14-01767-s001.zip › Table A5.pdf]

Table A5 | Primers designed for qRT-PCR.

Genes	Unigene ID	Forward primer sequence (5'-3')	Reverse primer sequence (5'-3')
Ef-1a	c97675_g4	CTGGGCACCGTGACTTTAT	TAGTGGCATCCATCTTGTT
DXR	c101158_g2	TGGACTAAAGCCTACAGTTGC	AAAGGACCACCAGCTATAAGAGT
DXS	c102923_g2	CTGTGAATGGTACTCTCCGAT	TTCCCCTTTTCTTACTGAACTCC
CMK	c97488_g1	CCTTTCTTCCGGATCGTACA	ACTGCCACAAACCCTTCATC
MCS	c88160_g1	TCTGTTTCTAATGGGTCGTC	TTAGAGCCTCATGCAACC
HDS	c105961_g2	TCATGGTTCAAGCCTATCGT	CCTCTCCAGCTTCAGTAACTCC
IDS	c104715_g2	AGAGATTCCTCCGTTTGCCA	CACAAAGACATGCGACCCAAC
AACT	c102830_g2	AATGGCAAAGTTGGAGTTGC	ACAGGCCATCACTTTGTTCC
HMGS	c102373_g1	TCAACAACCTAGATCAGTGCAA	TCTGTTTCCACGGTTTAGTC
HMGR	c89863_g1	CAACATGGCGAACAGTATGG	CTCGTGAAGAGCCACATGAA
PMK	c91944_g1	AATAGCCCCGAACAATCTGGT	TCCATGACTGAAGAAGCACAC
MVD	c89791_g1	GAAATGCCAATTACAGTAGCCAT	ACTTACATTGCCCATCGTCCA
IPI	c106536_g1	ATCAGAGTCCCCACAAAACGAA	TGTATCCCCTTCAGCGAGCAA
GPPS	c104431_g6	AGTTCTGGCGGTAGGCTATG	AGCCAGCGACAAGACCAC
SDR	c105839_g1	CAGCAAGTAAGCCAACGCCTA	TGAAATCCACAGCCCGACGAA
BPPS	c98804_g1	TAGAGCGGCTTGGTATTCAG	CCTGAGCAAGCGAAATCC
